# A Wavelet-Based Elevation Angle Selection Method for Soil Moisture Retrieval Using GNSS-IR

**DOI:** 10.3390/s25185609

**Published:** 2025-09-09

**Authors:** Xilong Kou, Yan Zhou, Qian Chen, Haigang Pang, Bo Sun

**Affiliations:** College of Information Science and Engineering, Shandong Agricultural University, Tai’an 271018, China; xilongkou@gmail.com (X.K.); zhouyansdust@163.com (Y.Z.); 17853538871@163.com (Q.C.); panghaigang11@gmail.com (H.P.)

**Keywords:** GNSS-IR, soil moisture, wavelet, elevation angle selection

## Abstract

Global Navigation Satellite System Interferometric Reflectometry (GNSS-IR) technology has emerged as a research hotspot in the remote sensing field in recent years due to its advantages of low cost and high precision for soil moisture monitoring. Addressing the issue that fixed elevation angle intervals struggle to adapt to the varying signal characteristics of different satellites, this paper proposes an adaptive elevation angle interval selection method based on wavelet transform. This method utilizes wavelet transform to analyze the time-frequency characteristics of the residual Signal-to-Noise Ratio (SNR) signal, calculates the ratio sequence of the main frequency component strength to the noise component strength, and sets a threshold to automatically determine the retrieval elevation angle interval for each satellite, thereby improving the accuracy of feature parameter extraction. The results show the following: ① Compared to traditional fixed elevation angle intervals (5–20° and 5–30°), the proposed method significantly enhances soil moisture retrieval accuracy. ② For the averaged phase feature parameters calculated within the algorithm-selected intervals for all satellites, the R^2^ and RMSE are 0.925 and 0.55%, respectively, representing improvements of 3.1% and 14.2% compared to the original results. ③ For signals from low-quality reflection zones, R^2^ increased from 0.728 to 0.839 (a 13.2% improvement), while RMSE decreased from 1.045 to 0.806 (a 22.9% reduction). This method effectively adapts to the quality attenuation characteristics of satellite signals across different reflection zones, providing an optimized elevation angle interval selection strategy for GNSS-IR soil moisture retrieval.

## 1. Introduction

Soil moisture is a key factor in land surface processes. By influencing surface energy and water exchange, it significantly impacts climate and the environment. Simultaneously, it plays a decisive role in vegetation growth and distribution, affecting the stability and diversity of entire ecosystems. It is an essential parameter in meteorology, ecology, agriculture, and related studies [1] GNSS-IR soil moisture retrieval technology offers advantages such as abundant signal sources, low cost, and high spatio-temporal resolution, holding significant research importance for providing soil moisture data to relevant industries and research.

Martin-Neira et al. (1993) [2] first proposed using reflected GPS signals for sea surface height measurement. Larson et al. (2008) [3] utilized the interference effect between direct and reflected signals causing SNR fluctuations to derive amplitude and phase, confirming their high correlation with soil moisture. This established the fundamental method of GNSS-IR technology. Zavorotny et al. (2010) [4] developed an interference physical model, simulating the impact of soil moisture changes on multipath interference phase and amplitude. Chew et al. (2014) [5] demonstrated that the multipath interference phase of the reflected signal is linearly correlated with surface soil moisture, making it the optimal parameter for soil moisture estimation. In subsequent work [6,7], they further validated the influence of vegetation on SNR morphology, which can reflect vegetation growth conditions around the specular reflection point. Han et al. (2018) [8] proposed a semi-empirical SNR model for soil moisture retrieval based on reconstructing direct and reflected signals from raw SNR data. Sun et al. (2020) [9] proposed a joint inversion method that weighted and fused L1, L2, and L5 band data from multiple GPS satellites, effectively improving retrieval accuracy. Chen et al. (2021) [10] proposed an improved soil moisture inversion algorithm based on principal component analysis (PCA) and an entropy method using multi-frequency amplitude and phase shift fusion data. PCA was applied to phase and amplitude, and the entropy method was used for fusion. He Jiaxing et al. (2023) [11] established a PSO-optimized CNN GNSS-IR soil moisture retrieval model by fusing multi-source observation data, which can effectively retrieve soil moisture and suppress the influence of different underlying surface environments to some extent. Li et al. (2023) [12] conducted simulation experiments on a layered model (vertical moisture variation) and employed Hilbert transform to obtain the interference signal envelope, reducing spike interference from environmental noise to improve retrieval accuracy, with plans for subsequent field data validation. Yao Jiang et al. (2024) [13] proposed a GNSS-IR soil moisture retrieval method based on multi-satellite data fusion using Random Forest (RF). This method utilizes the Mean Decrease in Impurity (MDI) algorithm of the RF model to adaptively assign weights to arcs, fusing all available satellite data to obtain accurate inversion results. Lili Jing et al. (2025) [14] proposed a soil moisture retrieval algorithm that effectively links the amplitude ratio of reflected to direct signals with an established model of reflectivity and dielectric properties, enabling the precise determination of soil moisture content.

Wavelet decomposition and related concepts are often applied to remove the trend term from SNR signals and distinguish between reflections from the ground and vegetation or water surface height [15]. Wu Haojian et al. (2022) [16] used wavelet decomposition transform instead of the traditional polynomial fitting method to obtain residual SNR and established a BP neural network model to invert soil moisture. Qin Ding et al. (2023) [17] employed Empirical Mode Decomposition (EMD) to replace the traditional low-order polynomial for SNR signal detrending, combined satellite selection using inter-satellite correlation coefficients, and finally introduced robust regression to enhance retrieval accuracy. Shekhar et al. (2023) [18] used wavelet analysis to extract low-frequency components from multipath signals, combined with the Lomb-Scargle Periodogram (LSP) to determine multipath frequency, analyzing its dependence on field soil moisture. Li et al. (2023) [19] separated reflected signals from the corn canopy and ground surface based on EMD, combined GNSS-IR altimetry to estimate canopy height, and used a semi-empirical microwave model to invert soil moisture. Wang Xiaolei et al. (2024) [20] applied wavelet analysis to GNSS-IR sea level height monitoring. The results demonstrated considerable consistency between the instantaneous frequencies extracted via wavelet analysis and those obtained from LSP analysis, though wavelet analysis still requires supplementary support from LSP.

Regarding the selection of the reflection elevation angle interval, existing algorithms typically choose a fixed interval. The values of this interval vary across different publications, mainly distributed within ranges such as 5–20° [21,22], 5–25° [23], and 5–30° [6,24]. However, these intervals may contain residual SNR patterns that deviate from the assumed cosine waveform due to undulating terrain, consequently affecting the results [25]. In fact, the values of feature parameters calculated within different elevation angle intervals often differ, leading to varying retrieval performance. For some signals, this difference can be substantial. Ran et al. (2022) [25] edited residual SNR arcs, retaining only those with typical cosine waveform interference patterns to improve the quality of the signals involved in fitting. Jiaxing He et al. (2025) [26] assessed the accuracy of curve fitting using the entropy method and employed the Particle Swarm Optimization (PSO) algorithm to identify SNR segments with typical waveforms from the residual SNR data, thereby improving result precision. However, the processing procedure is relatively complex. Therefore, this study proposes an adaptive elevation angle interval selection method based on wavelet transform. This method quantifies the time-varying spectral energy distribution of the residual SNR through wavelet time-frequency analysis, calculates the ratio of the main frequency band energy intensity to the non-main frequency band (noise) energy intensity, and adaptively determines the retrieval elevation angle interval for each satellite based on a preset energy threshold. This aims to maximize the utilization of effective signal components and suppress noise interference.

## 2. Materials and Methods

### 2.1. GNSS-IR Inversion Principle

The GNSS-IR technology primarily investigates the signal-to-noise ratio (SNR) under the interference of direct and reflected signals. Due to the relatively close positioning of GPS receivers to the ground, there exists a minimal time delay between direct and reflected signals at the receiver location. Within a single pseudo-random modulation code length, these signals can interfere freely and stably [4], with this interference phenomenon becoming more pronounced at low elevation angles [9]. Numerous existing studies have demonstrated that L-band electromagnetic waves utilized by navigation satellites exhibit superior sensitivity to soil moisture compared to other microwave bands, as well as penetration depths that closely align with those of ground-based soil moisture monitoring sensors [27]. The reflected signal carries characteristics related to the reflecting surface due to its interaction with media, which is then manifested in the SNR.

According to the principle of interference, the Signal-to-Noise Ratio (SNR) of the interference signal received by the antenna can be expressed as follows:

(1) $$SNR^{2}=S_{d}^{2}+S_{r}^{2}+2S_{d}S_{r}cos\psi$$

where *S_d_* and *S_r_* are the amplitudes of the direct and reflected signals, respectively, and *ψ* is the phase difference between the direct and reflected signals. Removing the trend term, the effect of the interference can be expressed as follows [21]:

(2) $$SNR_{m}=Acos\left(\frac{4\pi h}{\lambda }sin\theta +\varphi \right)$$

In the equation, *A* is the amplitude of the SNR residual sequence $SNR_{m}$, *h* is the distance from the antenna phase center to the ground, *λ* is the wavelength of the signal, *θ* is the satellite elevation angle, and φ is the phase difference. *h* is typically obtained through L-S (Lomb-Scargle Periodogram) spectral analysis. The frequency *f* corresponding to the maximum energy is taken, and is calculated using the following equation:

(3) $$f=\frac{2h}{\lambda }$$

The following is an empirical formula for calculating soil moisture using phase:

(4) $$SMC=K\left(\varphi -\varphi_{min}\right)+SMC_{resid}$$

where *K* is the slope between phase and soil moisture, $\varphi_{min}$ is the mean of the bottom 15% of phase values, and $SMC_{resid}$ is the residual moisture content.

**Figure 1:**
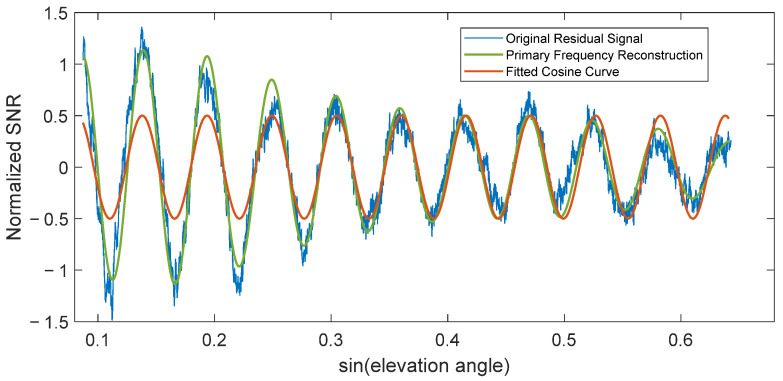
Standard Residual SNR and Fitted Curve.

In soil moisture inversion, *h* is typically taken as the average value over several days when its fluctuation remains relatively stable. By fitting the data using nonlinear least squares, the feature parameters A and φ are obtained. The extracted frequency f, amplitude A, or phase φ can all be used as feature parameters to invert ground physical properties [28]. Although *φ* is also influenced by factors such as surface cover, topography and roughness, and satellite orbit besides soil moisture, it exhibits good sensitivity to soil moisture [27,29] and strong correlation [30]. Therefore, this paper selects phase as the feature parameter. This study uses the main frequency component to reconstruct the signal after wavelet transform and fits the unknown parameter phase using the reconstructed signal. This can reduce noise influence to some extent. This method is used for all intervals in the subsequent sections. Figure 1 is a schematic diagram of fitting feature parameters using the residual SNR.

To reduce the impact of multipath effects on positioning accuracy, receiver antennas have low gain for reflected signals from high elevation angles, resulting in higher residual SNR intensity at low elevation angles. Additionally, the Fresnel reflection zone is larger at lower angles, smoothing small-scale surface inhomogeneities (rocks, minor undulations, etc.), which reduces sensitivity to changes in the reflection zone. This also makes the reflected signal of higher quality at low angles. Generally, the waveform of the residual SNR differs slightly among satellites, primarily due to differences in environmental factors. These include the topography and roughness of the reflection zone, reflections from other areas, the condition of the cover above the reflection zone, and satellite signal obstruction. If these conditions deviate significantly from the ideal, they can adversely affect the signal. For example, excessive terrain undulation in the reflection zone may prevent the residual SNR signal from forming a complete periodic fluctuation, rendering the model invalid and making it difficult to locate the actual reflection zone. Reflections from other areas (e.g., nearby buildings or mountains) can impose high-frequency fluctuations on the residual SNR signal. If the reflection zone has vegetation cover, it can weaken reflected signal strength and introduce vegetation interference [6]. Signals in high elevation angle intervals are more susceptible to environmental influences due to weaker reflected signal strength and smaller Fresnel reflection zone areas [31], with the specific impact location varying depending on the environment.

The feature parameters *A* and φ are obtained by fitting the discrete sequence values of the residual SNR within the selected interval using a cosine function. Different intervals correspond to different residual SNR sequences, resulting in different fitted parameter values. Expanding the interval provides more information but introduces more noise, necessitating a suitable interval. Furthermore, the attenuation rate of reflected signal quality varies depending on the reflection zone. Selecting a single fixed interval struggles to simultaneously adapt to the characteristics of different satellite signals. An algorithm is needed to find an appropriate interval for each satellite.

### 2.2. Wavelet Transform and Elevation Angle Interval Selection

Wavelet transform is a signal analysis method that decomposes a signal into components at different scales (frequencies) and locations. Unlike Fourier transform, which primarily focuses on frequency components, wavelet transform simultaneously considers frequency and time (or space) information, making it highly suitable for analyzing non-stationary time series where power and frequency change over time.

The core of wavelet transform is the wavelet function $\psi \left(t\right)$, an oscillatory function with finite length or rapid decay. The wavelet function is scaled and translated to analyze the signal. For the continuous wavelet transform, the wavelet function is $\psi_{a,b}\left(t\right)=\frac{1}{\sqrt{\left|a\right|}}\psi \left(\frac{t-b}{a}\right)$, where a is the scale factor and b is the translation factor. The continuous wavelet transform of a signal *x*(*t*) is defined as $W_{x}\left(a,b\right)=\int_{-\infty }^{\infty }\bar{x\left(t\right)\psi_{a,b}\left(t\right)}dt$, where $\bar{x\left(t\right)\psi_{a,b}\left(t\right)}$ is the complex conjugate of $x\left(t\right)\psi_{a,b}\left(t\right)$. The magnitude of the scale factor a affects the dilation of the wavelet function; a larger a stretches the wavelet function, corresponding to lower frequencies, while a smaller a compresses it, corresponding to higher frequencies. The translation factor b shifts the wavelet function along the time (or space) axis, enabling analysis at different signal positions [32].

Wavelet transform decomposes the signal into components at different resolutions (scales), providing information in both the time (or space) and frequency domains, and can precisely locate the time (or position) where a specific frequency component occurs. This property allows wavelet transform to assess the variation of signal strength with elevation angle at different scales, which is required for this study.

The phase feature parameter φ is essentially an inherent property of the residual SNR at its frequency f. Frequency bands far from f primarily contribute noise or secondary signals, which, if included in the fitting, would significantly contaminate the estimation of φ. Therefore, the elevation angle interval selection algorithm in this study calculates the main frequency signal strength and noise signal strength based on wavelet transform, computes their ratio, and determines the selection interval based on a threshold. This reduces the influence of low-quality signals and ensures consistency between the fitting data and the inherent assumption of the single-frequency cosine model (Equation (2)). This interval selection integrates main frequency strength and noise strength, automatically adapting to the characteristics of each satellite’s residual SNR, thereby improving signal utilization.

**Figure 2:**
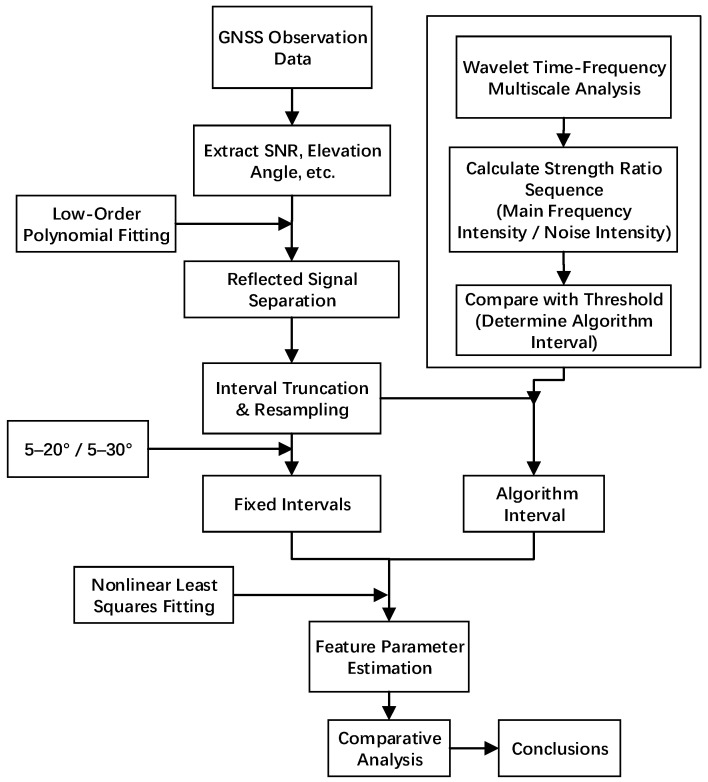
Flowchart.

Specific steps are as follows: (1) Process GNSS satellite observation files to extract necessary information such as elevation angle, SNR, and azimuth angle. (2) Fit the trend term using a low-order polynomial to obtain the residual SNR sequence. (3) Extract the residual SNR within the 5–40° elevation angle interval; subsequent interval selection will be performed within this range. (4) Resample the data using the sine of the elevation angle as the variable to achieve uniform sampling. (5) Perform wavelet transform on the resampled residual SNR signal, calculate the main frequency strength and noise strength at different elevation angles, and obtain the ratio sequence. (6) Set a threshold to obtain the maximum contiguous interval where the ratio exceeds the threshold; this interval is the selected interval. (7) Perform fitting to solve for the phase feature parameter. (8) Conduct comparative analysis with the original intervals. The flowchart is shown in Figure 2.

### 2.3. Data Collection and Acquisition

**Figure 3:**
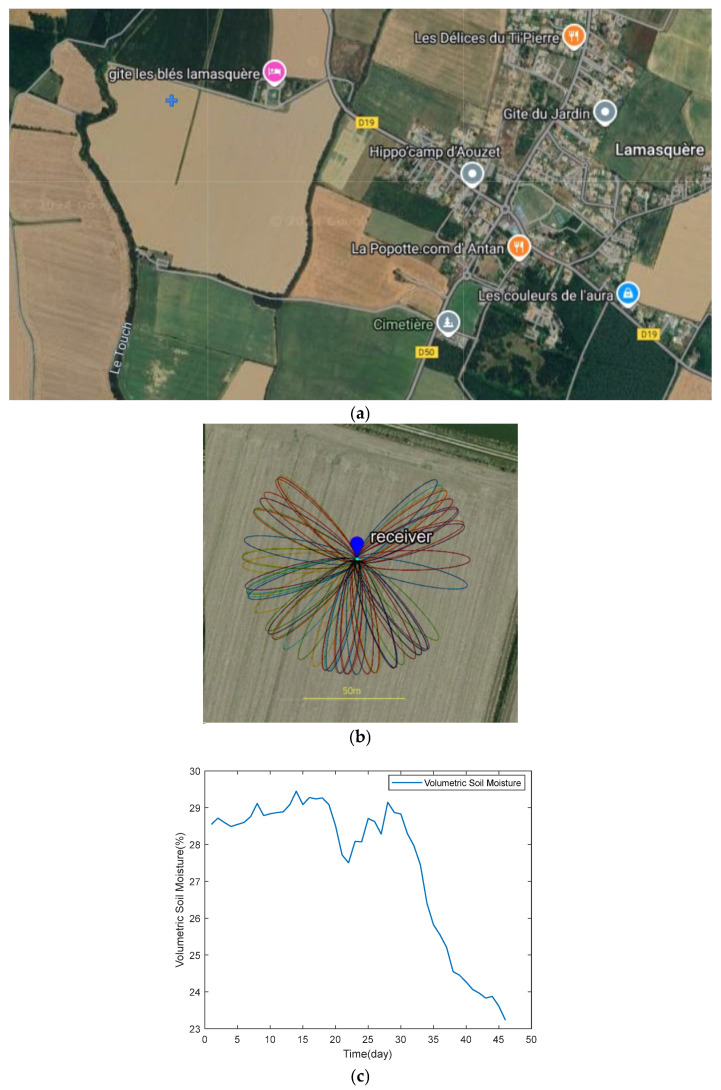
Overview of the Study Area: (**a**) Satellite map of the study area (blue crosses); (**b**) the Fresnel reflection zones (5 degrees) of each satellite orbit; (**c**) Reference value of soil moisture. Satellite basemap provided by Google Maps; Map data: © 2024 Google.

The experimental site is located in southwestern France, near the town of Lamasquère, close to Toulouse. This area is part of the research zone monitored by the CESBIO laboratory (UMR 5126) under the Sud-Ouest project [23]. The region has a mild climate, with temperatures remaining above 0 °C throughout the experiment. The average annual rainfall is approximately 600 mm. A Leica GR25 receiver and an AR10 antenna were installed in a cultivated field in Lamasquère, with the antenna positioned at a height of approximately 1.7 m. Data were continuously recorded at a frequency of 1 Hz for 46 days, from early February to mid-March 2014. The study area consists of bare land, providing an unobstructed environment around the antenna at close range. The terrain is flat with low roughness, characterized by a root mean square height of about 2 cm. Additionally, two soil moisture sensors were deployed on-site, each with an accuracy of 0.01 m^3^/m^3^ and positioned at depths of 2 cm and 5 cm, respectively. For this study, signals from the GPS L1 band with strong signal-to-noise ratio (SNR) characteristics [33] were selected for interferometric data analysis. The relevant details of the experimental area are illustrated in Figure 3: (a) the location of the study area, with the blue plus sign in the upper left corner indicating the position of the receiver; (b) the Fresnel reflection zones for different satellite orbits at a satellite elevation angle of 5 degrees. Since most satellites are concentrated near the equator and in low-latitude regions, there is typically a gap in the reflection zone towards the north. When setting up antennas for experiments in the Northern Hemisphere, they are usually placed on the northern side of agricultural fields to receive signals from the south. The situation is reversed in the Southern Hemisphere; and (c) the reference values of soil moisture during the study period. The satellite base map used in the figure is sourced from Google Maps (https://www.google.com/maps/, accessed on 10 May 2024).

The method outlined in reference [3] (the process of generating characteristic parameters in this technical approach) is employed to derive the time series of characteristic parameters. The specific steps are as follows: (1) Calculate essential information such as the SNR, elevation angle, and azimuth angle of GPS signals. This data is categorized into ascending and descending segments due to their differing reflection areas; for subsequent experiments, the ascending segment is selected. (2) Select data within the target elevation angle range (fixed or algorithm-determined). Remove the trend term using a low-order polynomial to obtain the residual SNR data. (3) Perform L-S spectral analysis on the fluctuation signals with respect to the sine value of the elevation angle as a variable to identify its dominant frequency. The median value will be taken as the final spectrum, and reference frequency will be calculated using Formula (3). Given that the experimental area is relatively flat, convert antenna height into frequency for determining dominant frequencies. (4) Once frequencies are established, utilize nonlinear least squares methods to determine parameters *A* and φ. Apply identical calculations across all satellite datasets to acquire sequences of characteristic parameters.

## 3. Results

This section describes the treatment processes and corresponding results of each part of the experiment.

**Figure 4:**
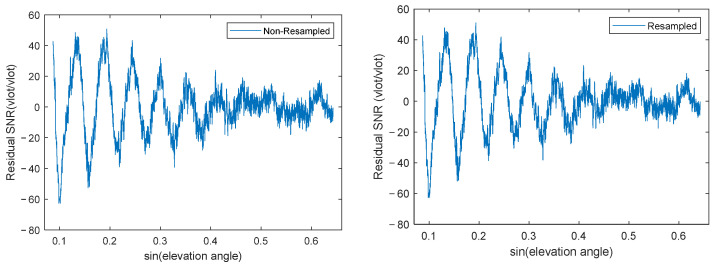
Residual SNR: (**Left**) Before resampling; (**Right**) After resampling.

Data preprocessing is performed first. Processing the GNSS satellite observation files yields sequences of elevation angles and corresponding SNR values. However, the elevation angles are not equally spaced, nor are their sine values, which adds difficulty to subsequent analysis. Therefore, a resampling step is required to convert the residual SNR data into uniformly sampled data. After detrending the SNR data and extracting the residual SNR signal within the 5–40° interval, the number of data points varies per satellite, typically between 4500 and 6000. Using the sine of the elevation angle as the variable, a polyphase anti-aliasing filter is used to resample the signal at a uniform sampling rate, resulting in 6500 uniformly distributed data points within this interval. Experimental verification showed that the correlation between the feature parameters calculated from the resampled data and soil moisture differed only minimally from the results using the original data. Figure 4 shows the residual SNR before and after resampling.

**Figure 5:**
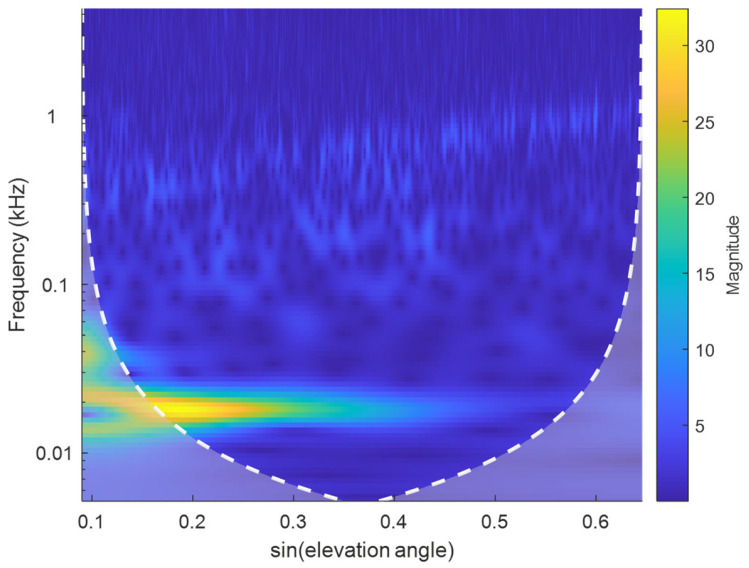
Time-frequency map of residual SNR.

Subsequently, wavelet transform is applied to the resampled data using the Morlet wavelet function, which derives from a combination of Gaussian and sinusoidal functions and is particularly well-suited for environmental analysis [21]. Frequencies are divided logarithmically, wavelet transform is performed, and the time-frequency map (Figure 5) of the residual SNR is evaluated.

**Figure 6:**
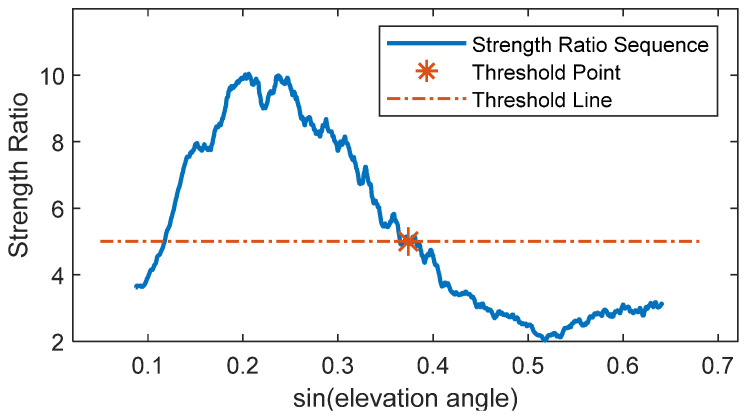
Strength Ratio Variation with Elevation Angle.

As shown in Figure 5 (Satellite 16), a bright band around 18 Hz indicates the main frequency band where signal energy is primarily concentrated. The brightness of this band decreases from left to right, indicating medium signal strength and attenuation rate for this satellite. Large areas outside this band are considered as noise; these signal components adversely affect feature parameter estimation. The wavelet’s peak frequency is identified, and the average energy intensity within ±3 scale frequencies around it is taken as the main frequency strength. The average energy intensity of other scale frequencies is calculated as the noise strength. Their ratio is then computed. The mean ratio sequence across different days is calculated for each satellite, yielding the elevation angle sequence of the strength ratio for that satellite, as shown in Figure 6.

**Figure 7:**
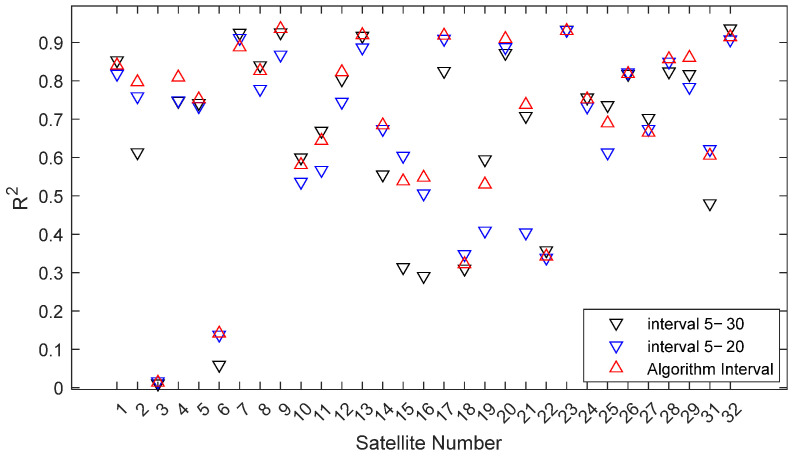
R^2^ Comparison of Different Intervals.

Figure 6 shows the ratio strength gradually decreasing. The beginning and end points lie outside the influence cone, where edge effects reduce data reliability, causing anomalies. Since residual SNR signal quality is generally better at low elevation angles, only the cut-off elevation angle needs selection to avoid mis-selection due to edge effects at the start. The attenuation threshold is determined experimentally; a threshold value of 5 yielded optimal results for the selected signal interval. Using this threshold, Satellite 16’s cut-off elevation angle is determined as 22°, forming a 5–22° selection interval (range preceding the red asterisk), while subsequent segments (region following the red asterisk below the threshold line) are discarded. The same process is applied to other satellites to obtain their corresponding elevation angle intervals, which are then used to solve for the phase feature parameter. For comparison, phase feature parameters are also solved using fixed intervals of 5–20° and 5–30°. Experimental results show that the correlation R^2^ between the phase feature parameters calculated by the proposed method and soil moisture is, for most satellites, greater than the maximum of the two fixed intervals, for some between them, and only rarely less than their minimum (Figure 7).

**Figure 8:**
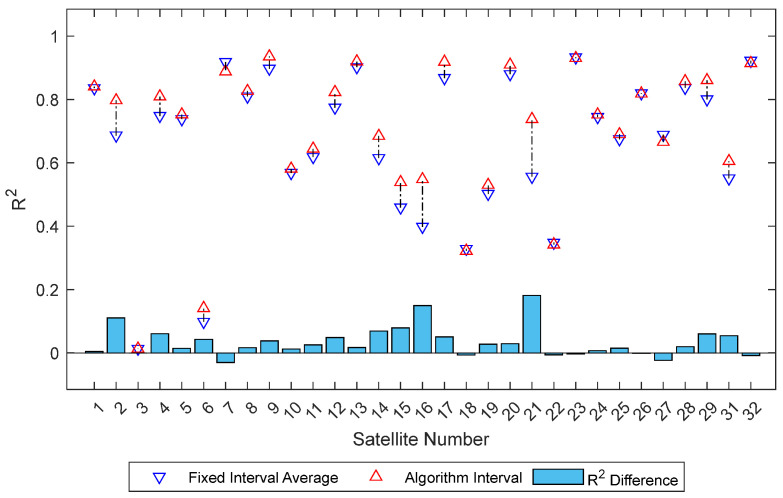
R^2^ Comparison: Algorithm Interval vs. Fixed Interval Average.

For ease of comparison, the average R^2^ and RMSE of the two fixed intervals are calculated. In Figure 8, the red upward triangles represent R^2^ calculated using the algorithm-selected intervals, while the blue downward triangles represent the average R^2^ of the two fixed intervals. The bar chart below shows the difference in R^2^ between the algorithm-selected interval and the fixed interval average for each satellite. The chart shows significant improvement for most satellites after interval selection by the algorithm, with only a few showing poorer performance.

**Figure 9:**
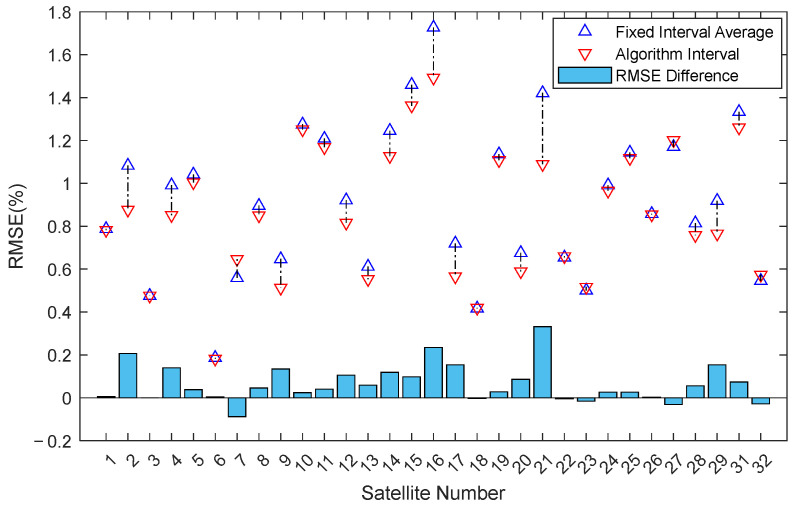
RMSE Comparison: Algorithm Interval vs. Fixed Interval Average.

Figure 9 shows a scatter plot of the Root Mean Square Error (RMSE) average for fixed intervals versus RMSE for algorithm-selected intervals, and the corresponding difference bar chart. Similarly, the algorithm-selected intervals show a significant advantage.

**Figure 10:**
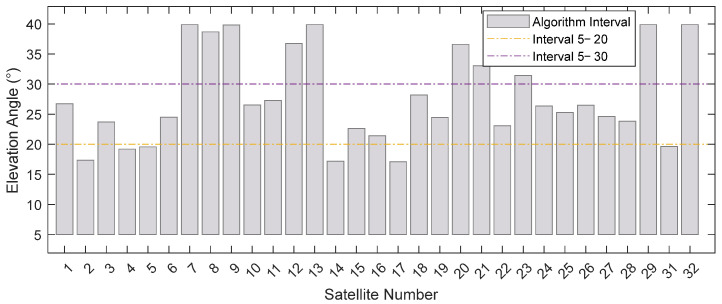
Distribution of algorithm-selected elevation angle intervals.

Figure 10 shows the distribution of elevation angle intervals selected by the algorithm. It demonstrates that the selected intervals are distributed across various ranges, indicating that a single fixed interval cannot adapt to this diversity.

**Figure 11:**
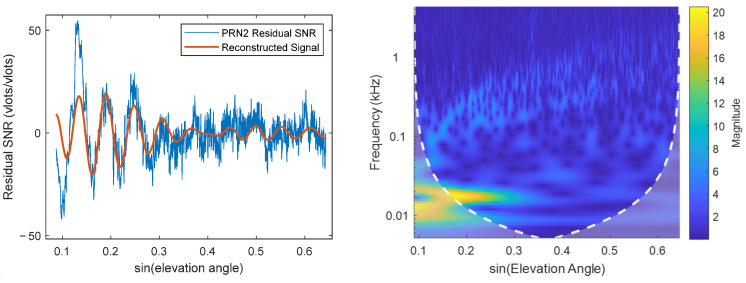
Satellite 2: (**Left**) Residual SNR; (**Right**) Wavelet time-frequency map.

**Figure 12:**
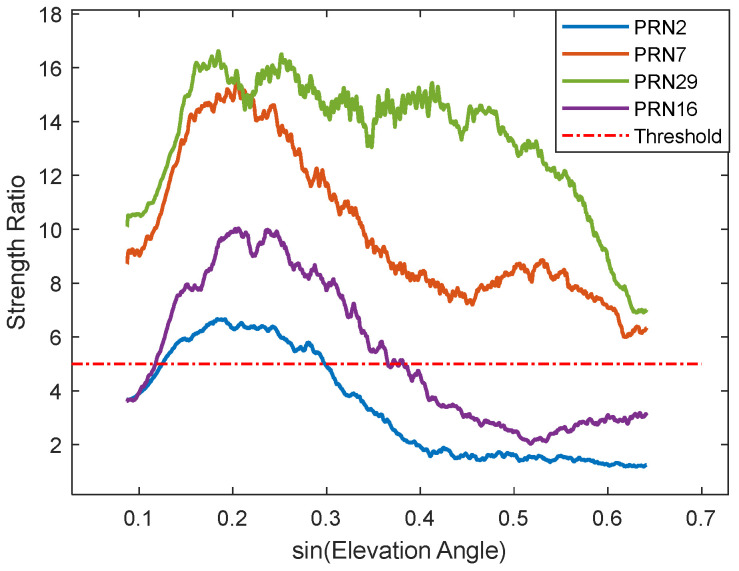
Strength Ratio Status of Various Satellites.

The residual SNR, wavelet time-frequency map (Figure 11), and ratio sequence (Figure 12) for Satellite 2 are plotted. Figure 11 shows that the signal quality deteriorates significantly after sine(elevation) ≈ 0.3, where a clear cosine waveform contour is difficult to discern. The strength of the main frequency component (red curve) in this region is very low, insufficient to resist noise influence. The time-frequency map also shows the main frequency strength rapidly attenuating with increasing elevation angle, becoming indistinguishable around sine(elevation) ≈ 0.4. This signal severely deviates from the model assumption and cannot provide useful information for parameter fitting, having a negative impact. Both fixed intervals (5–20° and 5–30°) include this noisy region, causing the feature parameter fitting to be significantly affected by noise, ultimately reducing accuracy. The algorithm calculated an interval of 5–17.4° for this satellite. Additionally, it was noted that the signal selection criteria of the algorithm are consistent with those used in [25]: (1) DSNR sequences with amplitudes less than 5 *v/v* were removed; (2) only high-quality DSNR data within specific elevation angle ranges were retained. Ultimately, the results obtained using the algorithm-selected interval outperformed those based on the original intervals, among which the fixed 5–30° interval showed the poorest performance.

**Figure 13:**
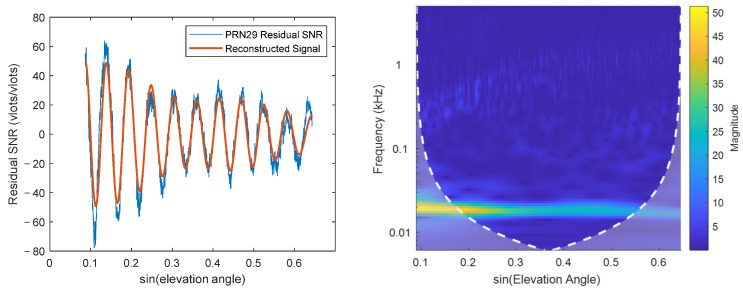
Satellite 29: (**Left**) Residual SNR; (**Right**) Wavelet time-frequency map.

Conversely, some satellites show poor results because the original fixed intervals were too short, occurring when the reflected signal maintains high strength even at higher elevation angles. The residual SNR, wavelet time-frequency map (Figure 13), and ratio sequence for Satellite 29 are plotted. Unlike Satellite 2, Satellite 29 exhibits high main frequency component strength throughout the 5–40° interval. Clear and strong fluctuation contours persist beyond 20° and 30°, providing useful information for parameter fitting. The ratio strength remains above the threshold of 5, leading to a selected interval of 5–40°. Results for this interval also showed improvement. Satellites like this include numbers 9, 13, etc.

Table 1 presents the average R^2^ and RMSE of the phase feature parameters calculated using the algorithm interval and different fixed intervals.

As shown in the table, the average R^2^ for the phase feature parameters using the algorithm interval is 0.925. This represents improvements of 2.1%, 4.1%, and 3.1% compared to the 5–20° fixed interval (0.906), the 5–30° fixed interval (0.889), and the fixed interval average (0.897), respectively. The RMSE is 0.551%, showing improvements of 10.2%, 17.6%, and 14.2% compared to the 5–20° fixed interval (0.614%), the 5–30° fixed interval (0.669%), and the fixed interval average (0.642%), respectively.

## 4. Discussion

**Figure 14:**
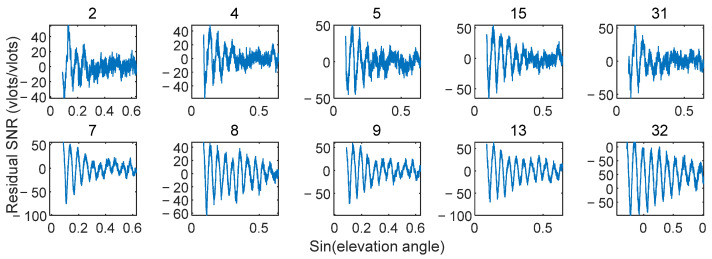
Residual SNR in Different Reflection Zones.

**Figure 15:**
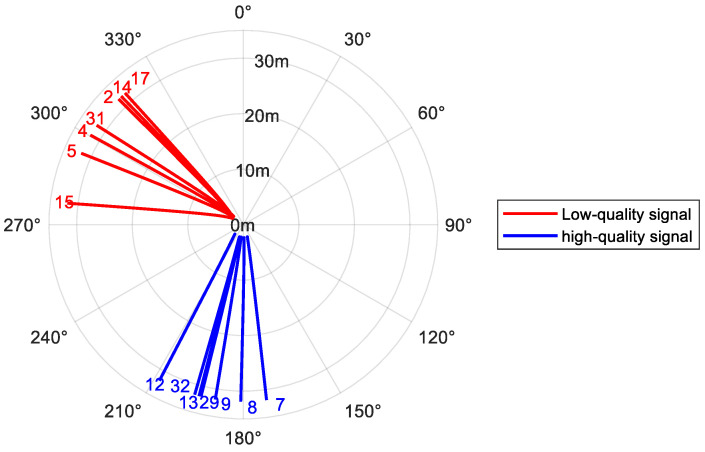
Track Distribution of Two Signal Reflection Zone Centers.

Satellites with short intervals (e.g., 2, 4, 5, 14, 15, 17, 31) and satellites with long intervals (e.g., 7, 8, 9, 12, 13, 29, 32) were summarized. Their residual SNR signals are shown in Figure 14. The latter group generally exhibited higher retrieval correlation and accuracy, while the former group had lower performance. This difference stems from the spatial heterogeneity of reflected signal quality with satellite azimuth and Fresnel reflection zone location. Figure 15 shows a schematic of the azimuth paths covered by satellites with long and short intervals. Although the experimental site was flat overall, unavoidable regional heterogeneity in surface characteristics (e.g., tillage marks, local ground undulations) existed spatially. This heterogeneity introduces regional effects on the reflected signal: areas that are closer to an ideal reflecting surface (e.g., the lower region in Figure 15) tend to produce clear interference patterns with high SNR (i.e., high-quality signal zones). In contrast, other areas (e.g., the left region in Figure 15) may exhibit greater surface roughness in that specific region or direction. Increased surface roughness enhances signal scattering and reduces the power of the reflected component, thereby leading to attenuated residual SNR signal strength and waveform distortion.

**Figure 16:**
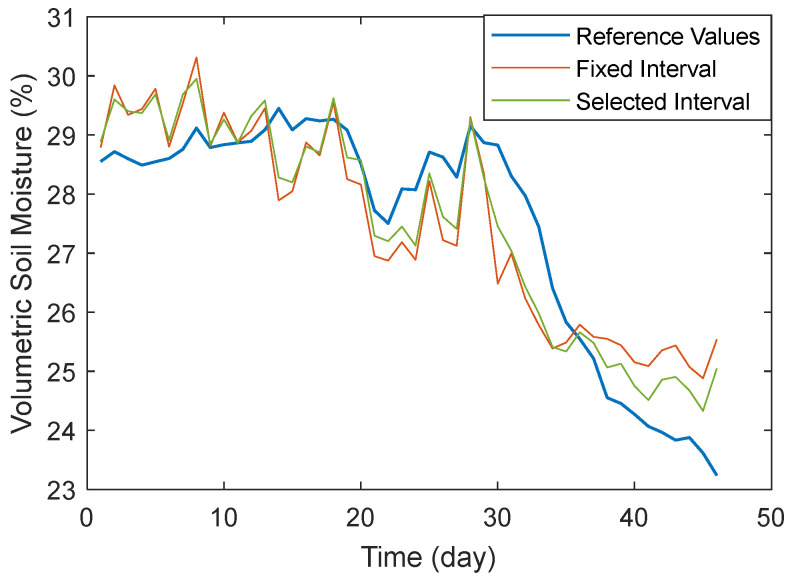
Comparison of the Performance of Different Algorithms.

The interval correction achieved more significant effects for signals from this low-quality zone. The average correlation R^2^ for signals in this zone increased from 0.728 to 0.839 (a 13.2% improvement), while RMSE decreased from 1.045 to 0.806 (a 22.9% reduction). Figure 16 shows a comparison between the mean results from the fixed interval and the algorithm-selected interval for satellites in this region. The results from the algorithm interval exhibit greater stability and closer alignment with reference values.

## 5. Conclusions

Selecting the fitting interval for feature parameters is a necessary step in GNSS-IR technology. Addressing the limitation of fixed intervals in adapting to different signal characteristics, this study utilized the ratio of main frequency strength to noise strength, calculated via wavelet transform, to evaluate signal quality at different elevation angles. This enables adaptive selection of the data interval for parameter estimation, improving the utilization of effective signal components. The principle was explained and experimentally analyzed, leading to the following conclusions:

For the average phase feature parameters of all satellites calculated within the algorithm-selected intervals, R^2^ reached 0.925, an improvement of 0.028 (3.1%) over the original fixed-interval average result. RMSE was 0.55%, an improvement of 14.2%.

For signals from low-quality reflection zones, R^2^ increased from 0.728 to 0.839 (a 13.2% improvement), while RMSE decreased from 1.045 to 0.806 (a 22.9% reduction).

## Figures and Tables

**Table 1:** Result Comparison of Different Intervals.

Metric	Algorithm Interval	5–20° Interval	5–30° Interval	Fixed Interval Average
R^2^	0.925	0.906	0.889	0.897
RMSE (%)	0.551	0.614	0.669	0.642

## Data Availability

The original contributions presented in the study are included in the article; further inquiries can be directed to the corresponding author.
